# Prediction of solid pseudopapillary tumor invasiveness of the pancreas based on multiphase contrast-enhanced CT radiomics nomogram

**DOI:** 10.3389/fonc.2025.1513193

**Published:** 2025-04-07

**Authors:** Dabin Ren, Liqiu Liu, Aiyun Sun, Yuguo Wei, Tingfan Wu, Yongtao Wang, Xiaxia He, Zishan Liu, Jie Zhu, Guoyu Wang

**Affiliations:** ^1^ Department of Radiology, Taizhou Central Hospital (Taizhou University Hospital), Taizhou, China; ^2^ CT Imaging Research Center, GE HealthCare, Shanghai, China; ^3^ Advanced Analytics, Global Medical Service, GE Healthcare, Hangzhou, China; ^4^ Central Research Institute, United Imaging Healthcare Group Co., Ltd, Shanghai, China; ^5^ Department of Radiology, Ningbo Medical Center LiHuiLi Hospital, Ningbo, China; ^6^ Clinical laboratory, Taizhou Central Hospital (Taizhou University Hospital), Taizhou, China

**Keywords:** contrast-enhanced computed tomography (CECT), radiomics, nomogram, pancreatic solid pseudopapillary neoplasm (PSPN), invasiveness

## Abstract

**Objectives:**

To construct a multiphase contrast-enhanced CT-based radiomics nomogram that combines traditional CT features and radiomics signature for predicting the invasiveness of pancreatic solid pseudopapillary neoplasm (PSPN).

**Methods:**

A total of 114 patients with surgical pathologic diagnoses of PSPN were retrospectively included and classified into training (n = 79) and validation sets (n = 35). Univariate and multivariate analyses were adopted for screening traditional CT features significantly associated with the invasiveness of PSPN as independent predictors, and a traditional CT model was established. Radiomics features were extracted from the contrast-enhanced CT images, and logistic regression analysis was employed to establish a machine learning model, including an unenhanced model (model U), an arterial phase model (model A), a venous phase model (model V), and a combined radiomics model (model U+A+V). A radiomics nomogram was subsequently constructed and visualized by combining traditional CT independent predictors and radiomics signature. Model performance was assessed through Delong’s test and receiver operating characteristic (ROC) curve analysis. Decision curve analysis (DCA) was applied to assess the model’s clinical utility.

**Results:**

Multivariate analysis suggested that solid tumors (OR = 6.565, 95% CI: 1.238–34.816, P = 0.027) and ill-defined tumor margins (OR = 2.442, 95% CI: 1.038–5.741, P = 0.041) were independent predictors of the invasiveness of PSPN. The areas under the curve (AUCs) of the traditional CT model in the training and validation sets were 0.653 and 0.797, respectively. Among the four radiomics models, the model U+A+V exhibited the best diagnostic performance, with AUCs of 0.857 and 0.839 in the training and validation sets, respectively. In addition, the AUCs of the nomogram in the training and validation sets were 0.87 and 0.867, respectively, which were better than those of the radiomics model and the traditional CT model. The DCA results indicated that with the threshold probability being within the relevant range, the radiomics nomogram offered an increased net benefit to clinical decision making.

**Conclusion:**

Multiphase contrast-enhanced CT radiomics can noninvasively predict the invasiveness of PSPN. In addition, the radiomics nomogram combining radiomics signature and traditional CT signs can further improve classification ability.

## 1 Introduction

Pancreatic solid pseudopapillary neoplasm (PSPN) is a rare, low-grade malignant tumor accounting for 0.9% – 2.7% of all pancreatic tumors. Previous studies have shown that PSPN is more common in women under 40 years of age, whereas in men, the incidence of PSPN is lower, the age of onset is much older, and the malignant grade is higher (1–5). PSPN is a heterogeneous tumor, with a minor subset potentially exhibiting invasive characteristics, which significantly influence patient prognosis. Invasive behavior may involve perineural invasion, infiltration of adjacent organs and blood vessels, invasion into the pancreas and surrounding adipose tissue, as well as distant metastasis and regional lymph node involvement (6, 7). Currently, surgical resection is considered the preferred and most effective treatment for PSPN, and radical surgery is associated with a favorable prognosis, achieving a postoperative survival rate of 80% – 90% (8–10). In recent years, conservative surgical methods, such as tumor exenteration or laparoscopic surgery, have often been used for noninvasive PSPN patients, whereas for invasive PSPN patients, extended radical resection is needed to ensure a satisfactory long-term prognosis, and incomplete resection or positive resection margins may increase the risk of recurrence (11–13). Compared with conservative surgical methods, a wide range of pancreatic resections may lead to postoperative complications and pancreatic endocrine and exocrine dysfunction. Consequently, the preoperative differentiation of invasive and noninvasive PSPN is highly important for selecting appropriate surgical methods in the clinic and avoiding unnecessary resection (14, 15). However, obtaining preoperative pathological results is often challenging. The accuracy of pathological diagnosis from needle biopsy is restricted by the sample quality and quantity, which may not adequately reflect tumor heterogeneity. Additionally, needle biopsy carries the risk of tumor cell dissemination along the needle tract. Consequently, this complicates and raises controversy regarding the selection of the appropriate surgical approach for surgeons (16, 17).

CT is the first choice for the examination of pancreatic lesions, and enhanced CT can display invasive signs well, which is highly valuable for the preoperative evaluation of invasive and noninvasive PSPN (2). Nevertheless, image interpretation is limited by the subjectivity of radiologists; thus, finding a quantitative and objective method to evaluate invasive and noninvasive PSPN is highly important. Radiomics uses high-throughput extraction of implicit quantitative features (texture, shape, wavelet transform parameters, etc.) in images, combined with machine learning algorithms to mine tumor heterogeneity information, providing a new idea for non-invasive prediction of PSPN invasiveness. Previous studies have shown that radiomics has great potential in the diagnosis, assessment of invasiveness, biochemical recurrence prediction, and metastasis of pancreatic tumors (18–21). Tobaly et al. (18) found that radiomics features extracted from CT images can distinguish low-risk and high-risk Intraductal papillary mucinous neoplasms and guide surgical decision-making. Shi et al. (20) extracted radiomics features based on MR images and constructed a model combining clinical information to differentiate PSPN and pancreatic neuroendocrine tumor. The study results showed that the accuracy of the radiomics model was 92.42%, which was significantly higher than that of subjective diagnosis.

However, previous studies have focused on differentiating PSPN from other pancreatic tumors. Few studies have employed contrast-enhanced CT (CECT) radiomics to predict the invasiveness of PSPN. The purpose of this study was to developed and demonstrated a radiomics nomogram based on radiomics and traditional CT signs to predict the invasiveness of PSPN and provide clinicians with treatment decisions, especially the choice of surgical approach.

## 2 Materials and methods

### 2.1 Participants

**Figure 1:**
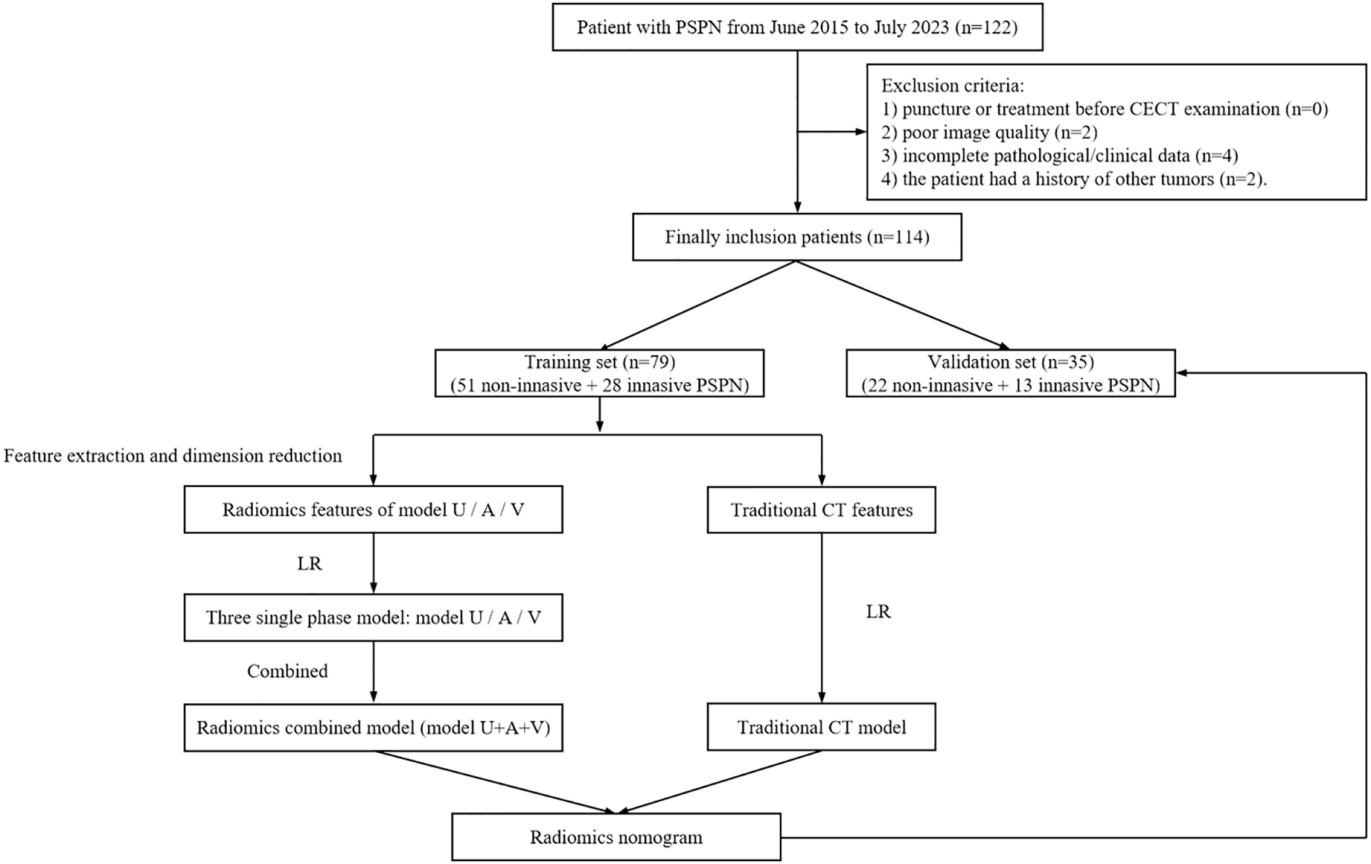
Study flow chart. PSPN, pancreatic solid pseudopapillary neoplasm; CECT, contrast-enhanced computed tomography; LR, logistic regression; Model U, model based on unenhanced CT; Model A, model based on arterial phase CT; Model V, model based on venous phase CT.

This study was approved by the Ethics Committee of Taizhou central hospital (Approval Number: 2024L-07-20) and Ningbo medical central Lihuili hospital (Approval Number: KY2024SL370-01), and the requirement of informed consent was waived due to the retrospective nature of this two-center study. Patients with a pathological diagnosis of PSPN were retrospectively collected between June 2015 and July 2023 according to the following inclusion criteria: (I) without a history of additional tumor types; (II) underwent CECT before surgery (including unenhanced scanning, arterial phase, and venous phase enhanced scanning); and (III) underwent surgery within 30 days after CECT examination. The exclusion criteria were as follows: (I) the patient had undergone puncture or treatment before CECT examination; (II) poor image quality; and (III) incomplete pathological/clinical data. The patient recruitment process is shown in **Figure 1**. A total of 114 PSNP cases were included in the present study, 42 of whom were from Taizhou central hospital and the remaining were from Ningbo medical central Lihuili hospital.

### 2.2 CT examination protocol

Patients were scanned using 64-row CT (Discovery CT 750 HD, GE Healthcare, Waukesha, WI, United States). Unenhanced scanning and dual-phase enhanced scanning were performed while the patients were in the supine position with their breath held. For contrast-enhanced examination, iohexol (Omnipaque, 350 mgI/mL, GE Healthcare) was injected via the superior cubital vein at a rate of 2.5 – 3.0 mL/s and a dose of 1.5 – 2.0 mL/kg. In addition, arterial and venous phase enhanced images were acquired at about 25–30 s and 50–60 s post-injection respectively. The CT parameters of the two medical centers are detailed in **Table 1**.

**Table 1:** CT parameters of the two medical centers.

Parameter	Medical center A	Medical center B
No. of rows	64	64
Tube voltage (kV)	120	120
Tube current (mA)	300	250
Slice thickness (mm)	1	1
Slice interval (mm)	1	1
Detector collimation (mm)	0.75	0.625
Rotation time (s)	0.5	0.5
Matrix	512 × 512	512 × 512

### 2.3 Analysis of images

The CT images were analyzed by 2 radiologists with more than 10 years of experience in abdominal diagnosis, and when there was disagreement, it was negotiated.

A qualitative evaluation revealed the following: tumor location (pancreatic head, neck, body and tail), tumor shape (round, irregular), tumor margin (well-defined, ill-defined), the presence of calcification, pancreatic atrophy, and pancreatic duct dilatation.

Quantitative evaluation: Manual measurements were performed 3 times, and the average value was calculated as follows: (I) the maximum diameter of the tumor; (II) tumor texture: a cystic area less than 30% was defined as a solid lesion, a cystic area greater than 70% was defined as a cystic lesion, and the remaining proportion was defined as a mixed cystic-solid lesion. The cystic components were defined as those with CT attenuation < 20 Hounsfeld Units on unenhanced images.

### 2.4 Pathology analysis

All pathological results were derived from postoperative biopsies and subsequently re-evaluated by a pathologist with 15 years of experience. Invasive characteristics of PSPN encompass infiltration into peripancreatic fat, pancreatic parenchyma, peripheral nerves, and vascular walls, as well as metastasis to adjacent organs, lymph nodes, and distant sites. In the absence of these features, the PSPN was classified as noninvasive.

### 2.5 Lesion segmentation and feature extraction

**Figure 2:**
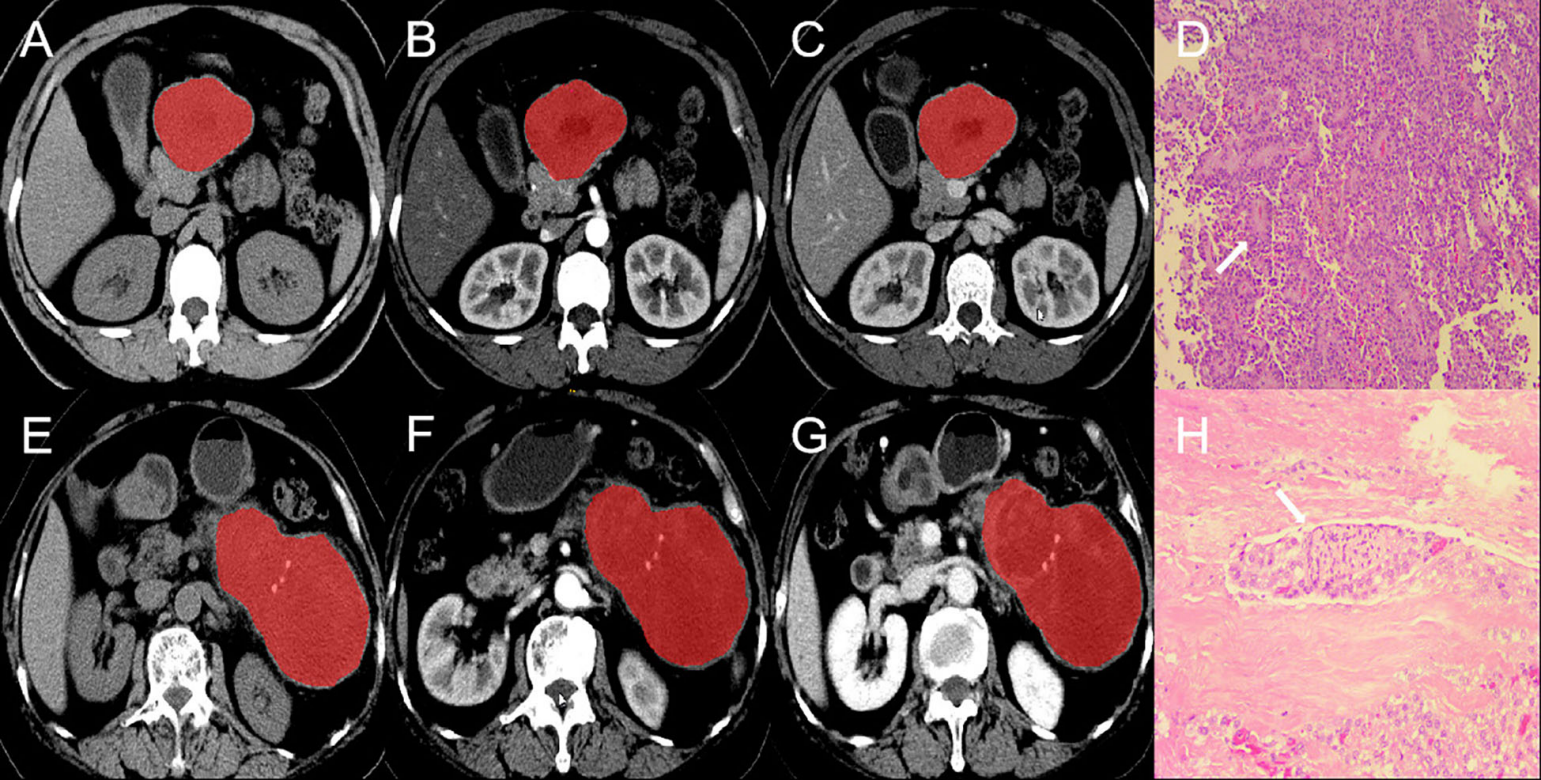
ROI segmentation and pathological section of invasive and non-invasive PSPN. **(A–C)** are the images of unenhanced phase **(A)**, arterial phase **(B)** and venous phase **(C)** of a case of non-invasive PSPN. **(D)** showed that the tumor was arranged in lamellar pattern and pseudopapillary structures (arrow). Tumor cells are composed of round or oval cells with uniform chromatin, inconspicuous or small nucleoli, cytoplasm eosinophilic, and occasional vacuolization (HE × 10). **(E–G)** are the images of unenhanced phase **(D)**, arterial phase **(E)** and venous phase **(F)** of a case of invasive PSPN. **(H)** showed that the shows tumor cells surrounding the nerve (arrow), indicating nerve invasion (HE × 10). ROI, region of interest. PSPN, pancreatic solid pseudopapillary neoplasm.

All CT images of the largest section of the lesion were imported into ITK-SNAP (https://www.nitrc.org/projects/itk-snap/) in Dicom format. All the images were preprocessed before the region of interest (ROI) was delineated to reduce differences in the images collected by different scanners. The CT images were resampled to a voxel size of 1 × 1 × 1 mm^3^ to normalize the voxel spacing and underwent gray-level discretization. Without knowledge of the patient’s clinical information and pathological results, a radiologist with 10 years of experience in abdominal diagnostic radiology manually delineated the ROI (**Figure 2**). The Pyradiomics package (https://github.com/radiomics/pyradiomics) in Python was utilized for extracting radiomics features, including (I) first-order features; (II) shape features; (III) second-order features originating from the gray-level run-length matrix (GLRLM), gray-level cooccurrence matrix (GLCM), gray-level dependence matrix (GLDM), neighborhood gray-tone difference matrix (NGTDM), and gray-level size zone matrix (GLSZM). (IV) High-order statistical features obtained via the wavelet transform of the original image.

To assess the reproducibility radiomics features, 30 patients were randomly chosen, and intra- and interobserver consistency was assessed by radiologist A (with 10 years of experience) and radiologist B (with 5 years of experience), both blinded to patient information. Radiologist A employed the same method to perform two separate ROI delineations and radiomics feature extractions for the same patient within one week. Radiologist B independently delineated the ROI and extracted features, which were then compared with the features obtained by radiologist A during the initial assessment. The intra- and interobserver reproducibility of radiomics feature extraction was assessed with the interclass correlation coefficient (ICC). An ICC ≥ 0.75 was considered to indicate high agreement between the ROIs delineated by radiologists A and B.

### 2.6 Radiomics feature selection and model construction

All invasive and noninvasive PSPN patients were randomized into training (70%) and validation (30%) sets. Radiomics features with high reproducibility were screened out with the interclass correlation coefficient. To ensure the uniformity of the feature scale, all radiomics features were standardized with z-score normalization. The remaining features were assessed using constant term elimination and Spearman’s correlation analysis, applying a threshold of r ≥ 0.8 to exclude features exhibiting high covariance. The least absolute shrinkage and selection operator (LASSO) method was then employed to reduce the dimensionality of the remaining features. A 5-fold cross-validation technique was utilized to select the tuning parameter (λ) in the LASSO model within the training set. The λ value was optimized to minimize binomial deviance, allowing for the selection of the most effective features. Finally, the radiomics score (Rad-score) was calculated by a linear combination of selected features, and the calculation formula of the Rad-score is presented in **Supplementary Figure S1**.

Univariate and multivariate analyses were adopted for screening traditional CT features significantly associated with the invasiveness of PSPN as independent predictors, and a traditional CT model was established. Four radiomics models were constructed on the basis of the Rad-score with logistic regression: an unenhanced model (model U), an arterial phase model (model A), a venous phase model (model V), and a radiomics combined model (model U+A+V). Finally, a radiomics nomogram was constructed by combining traditional independent CT predictors and the Rad-score of the combined radiomics model.

### 2.7 Statistical analysis

SPSS (https://www.ibm.com/products/spssstatistics, version 25) and R software (https://www.r-project.org/, version 4.0.4) were used for the statistical analysis. Continuous variables are presented as the means ± standard deviations (SD), and categorical variables are presented as frequencies and percentages. The normality of continuous variables was tested using the Kolmogorov-Smirnov test. Normally distributed continuous variables were explored using t-test, whereas the Mann-Whitney U test was used to compare nonnormally distributed variables. Categorical variables were tested using Fisher’s exact test or chi-square test. The calibration curve and Hosmer-Lemeshow test were employed to evaluate the calibration and goodness of fit of the nomogram. Model diagnostic efficacy was evaluated through receiver operating characteristic (ROC) curves. Delong’s test was applied to evaluate significant differences between AUCs. Finally, decision curve analysis (DCA) was performed to assess the model’s clinical utility. *P* < 0.05 represented statistical significance.

## 3 Results

### 3.1 Demographic and traditional CT feature analysis

All patients were randomized into training (n = 79) and validation (n = 35) sets. Among them, 36.0% of patients were classified as invasive PSPN (n = 41), and 64.0% of patients were classified as noninvasive PSPN (n = 73). All 114 patients underwent surgical resection, and the following instances of invasion were observed: the pancreas (n = 11), neural bundle (n = 11), surrounding fat (n = 4), blood vessels (n = 1), spleen (n = 2), common bile duct (n = 1), and multiple sites (n = 11). Tumor texture and margins were significantly different between invasive and noninvasive PSPN patients (*P* < 0.05), whereas location, diameter, shape, margins, presence of calcification, pancreatic atrophy, and pancreatic duct dilation were not significantly different (*P* > 0.05). Complete clinical and imaging information is detailed in **Table 2**. There was no significant difference between the clinical information and the traditional CT features in the training set (*P* > 0.05), whereas the tumor diameter and texture were significantly different in the validation set (*P* < 0.05), as displayed in **Table 3**.

**Table 2:** Clinical information and traditional CT features of 114 PSPN.

Features	Total (n=114)	Invasive PSPN (n=41)	Non-invasive PSPN (n=73)	*P* value
Sex				
Male	27 (23.7)	11 (26.8)	16 (21.9)	0.647
Female	87 (76.3)	30 (73.2)	57 (78.1)	
Age	36.55 ± 12.71	37.34 ± 14.43	36.11 ± 11.72	0.622
Location				
Head/Neck	50 (43.9)	16 (39.0)	34 (46.6)	0.711
Body/Tail	64 (56.1)	25 (61.0)	39 (53.4)	
Shape				
Round	89 (78.1)	33 (80.5)	56 (76.7)	0.814
Irregular	25 (21.9)	8 (19.5)	17 (23.3)	
Diameter	4.66 ± 2.55	4.93 ± 2.70	4.051 ± 2.48	0.502
Texture				
Solid	31 (27.2)	17 (41.4)	14 (19.2)	0.011^*^
Cystic	16 (14.0)	2 (4.9)	14 (19.2)	
Mixed cystic-solid	67 (58.8)	22 (53.7)	45 (61.6)	
Calcification	53 (46.5)	19 (46.3)	34 (46.6)	1.000
Margin				
Well-defined	78 (68.4)	22 (53.7)	56 (76.7)	0.013^*^
Ill-defined	36 (31.6)	19 (46.3)	17 (23.3)	
Dilation of pancreatic duct	3 (2.6)	0	3 (4.1)	0.480
Pancreatic atrophy	17 (14.9)	5 (12.2)	12 (16.4)	0.597

Categorical variables shown with frequency and percentage; continuous variables shown with mean ± standard deviation (SD); PSPN, pancreatic solid pseudopapillary neoplasm; ^*^P<0.05.

**Table 3:** Traditional CT features and Rad-score of models in training and validation sets.

Variables	Training set (n=79)				Validation set (n=35)			
	Non-innasive (n=51)	Innasive (n=28)	*t/Z/χ^2^*	P value	Non-innasive (n=22)	Innasive (n=13)	*t/Z/χ^2^*	P value
Sex								
male	10 (19.6)	9 (32.1)	1.555	0.273	6 (27.3)	2 (15.4)	-	0.680
female	41 (80.4)	19 (67.9)			16 (72.7)	11 (84.6)		
Age	35.7 ± 10.65	36.89 ± 14.21	-0.303	0.766	36.91 ± 14.12	38.31 ± 15.41	-0.154	0.886
Location								
head/neck	23 (45.1)	14 (50.0)	0.174	0.814	11 (50.0)	2 (15.4)	-	0.070
body/tail	28 (54.9)	14 (50.0)			11 (50.0)	11 (84.6)		
Shape								
round	38 (74.5)	23 (82.1)	0.599	0.578	18 (81.8)	10 (76.9)	-	1.000
irregular	13 (25.5)	5 (17.9)			4 (18.2)	3 (23.1)		
Diameter	4.91 ± 2.68	4.82 ± 2.98	-0.580	0.566	3.591 ± 1.62	5.15 ± 2.03	-2.241	0.024^*^
Texture								
solid	12 (23.5)	11 (39.3)	3.061	0.212	2 (9.1)	6 (46.2)	-	0.016^*^
cystic	9 (17.7)	2 (7.1)			5 (22.7)	0		
cystic-solid	30 (58.8)	15 (53.6)			15 (68.2)	7 (53.8)		
Calcification	25 (49.0)	16 (57.1)	0.478	0.638	9 (40.9)	3 (23.1)		0.463
Margin								
well-defined	38 (74.5)	16 (57.1)	2.520	0.134	18 (81.8)	6 (46.2)	-	0.057
ill-defined	13 (25.5)	12 (42.9)			4 (18.2)	7 (53.8)		
Dilation of pancreatic duct	2 (3.9)	0	1.127	0.537	1 (4.5)	0	-	1.000
Pancreatic atrophy	8 (15.7)	4 (14.3)	0.028	1.000	4 (18.2)	1 (7.6)	-	0.630
Rad-score								
Rad-score_U	-0.23 ± 1.04	0.43 ± 1.07	-2.675	0.007^*^	0.05 ± 1.55	1.13 ± 1.10	-2.255	0.031^*^
Rad-score_A	-0.24 ± 0.92	0.67 ± 0.78	-4.059	0.001^*^	-0.07 ± 1.39	1.07 ± 1.15	-2.629	0.007^*^
Rad-score_V	-0.12 ± 0.62	0.43 ± 0.53	-3.639	0.001^*^	-0.38 ± 0.77	0.26 ± 0.64	-2.356	0.017^*^
Rad-score_U+A+V	-0.96 ± 0.93	0.32 ± 0.77	-5.227	0.001^*^	0.01 ± 1.38	1.72 ± 1.14	-3.312	0.001^*^

Categorical variables shown with frequency and percentage; continuous variables shown with mean ± standard deviation (SD); Rad-score, radiomics score; U, unenhanced CT; A, arterial phase CT; V, venous phase CT. *P<0.05.

### 3.2 Traditional CT model construction

Univariate logistic regression analysis revealed that a solid tumor (OR = 0.850, 95% CI: 1.646–43.897, *P* = 0.011) and an ill-defined tumor margin (OR = 2.845, 95% CI: 1.254–6.455, *P* = 0.012) were significantly related to the invasion of PSPN, whereas other traditional CT features were not statistically significant. Subsequent multivariate analysis revealed that solid tumors (OR = 6.565, 95% CI: 1.238–34.816, P = 0.027) and ill-defined tumor margins (OR = 2.442, 95% CI: 1.038–5.741, P = 0.041) were independent predictors of the invasiveness of PSPN, as detailed in **Table 4**. The traditional CT model was constructed with solid tumors and ill-defined tumor margins as independent predictors, and its AUCs in the training and validation sets were 0.653 and 0.797, respectively (**Table 5**).

**Table 4:** Univariate and multivariate analysis of clinical information and traditional CT features.

Variables	Univariate analysis		Multivariate analysis	
	OR (95% CI)	P value	OR (95% CI)	*P* value
Sex	1.306 (0.539–3.168)	0.554		
Age	1.008 (0.978–1.039)	0.618		
Diameter	1.065 (0.919–1.235)	0.403		
Shape	0.799 (0.311–2.053)	0.641		
Location	1.362 (0.626–2.966)	0.436		
Texture	-	0.018	-	0.036
cystic-solid	3.422 (0.714–16.398)	0.124	2.640 (0.535–13.033)	0.234
solid	8.500 (1.646–43.897)	0.011	6.565 (1.238–34.816)	0.027
Calcification	0.991 (0.460–2.133)	0.981		
Margin	2.845 (1.254–6.455)	0.012	2.442 (1.038–5.741)	0.041
Dilation of pancreatic duct	-	0.999		
Pancreatic atrophy	0.706 (0.230–2.167)	0.543		

OR, odds ratio; CI, confidence interval.

**Table 5:** Diagnostic efficacy of all models in training and validation sets.

Model	AUC (95%CI)	Accuracy	Sensitivity	Specificity	PPV	NPV
Training set						
Single-phase radiomics model						
Model U	0.683 (0.575–0.791)	0.696	0.500	0.804	0.583	0.745
Model A	0.777 (0.683–0.862)	0.759	0.571	0.863	0.696	0.786
Model V	0.749 (0.649–0.836)	0.722	0.679	0.745	0.594	0.809
Radiomics combined model (model U+A+V)	0.857 (0.778–0.925)	0.797	0.786	0.804	0.668	0.872
Traditional CT model	0.653 (0.553–0.749)	0.633	0.679	0.608	0.487	0.775
Radiomics nomogram	0.874 (0.804–0.933)	0.823	0.714	0.882	0.769	0.849
Validation set						
Single-phase radiomics model						
Model U	0.699 (0.543–0.852)	0.571	0.692	0.500	0.450	0.733
Model A	0.769 (0.625–0.905)	0.686	0.692	0.682	0.562	0.789
Model V	0.741 (0.584–0.892)	0.771	0.538	0.909	0.778	0.769
Radiomics combined model (model U+A+V)	0.839 (0.716–0.944)	0.629	1.000	0.409	0.500	1.000
Traditional CT model	0.797 (0.674–0.910)	0.771	0.769	0.773	0.667	0.850
Radiomics nomogram	0.867 (0.759–0.959)	0.829	0.923	0.773	0.706	0.944

AUC, area under curve; CI, confidence interval; PPV, positive predictive value; NPV, negative predictive value; Model U, model based on unenhanced CT; Model A, model based on arterial phase CT; Model V, model based on venous phase CT.

### 3.3 Feature selection and radiomics model construction

**Figure 3:**
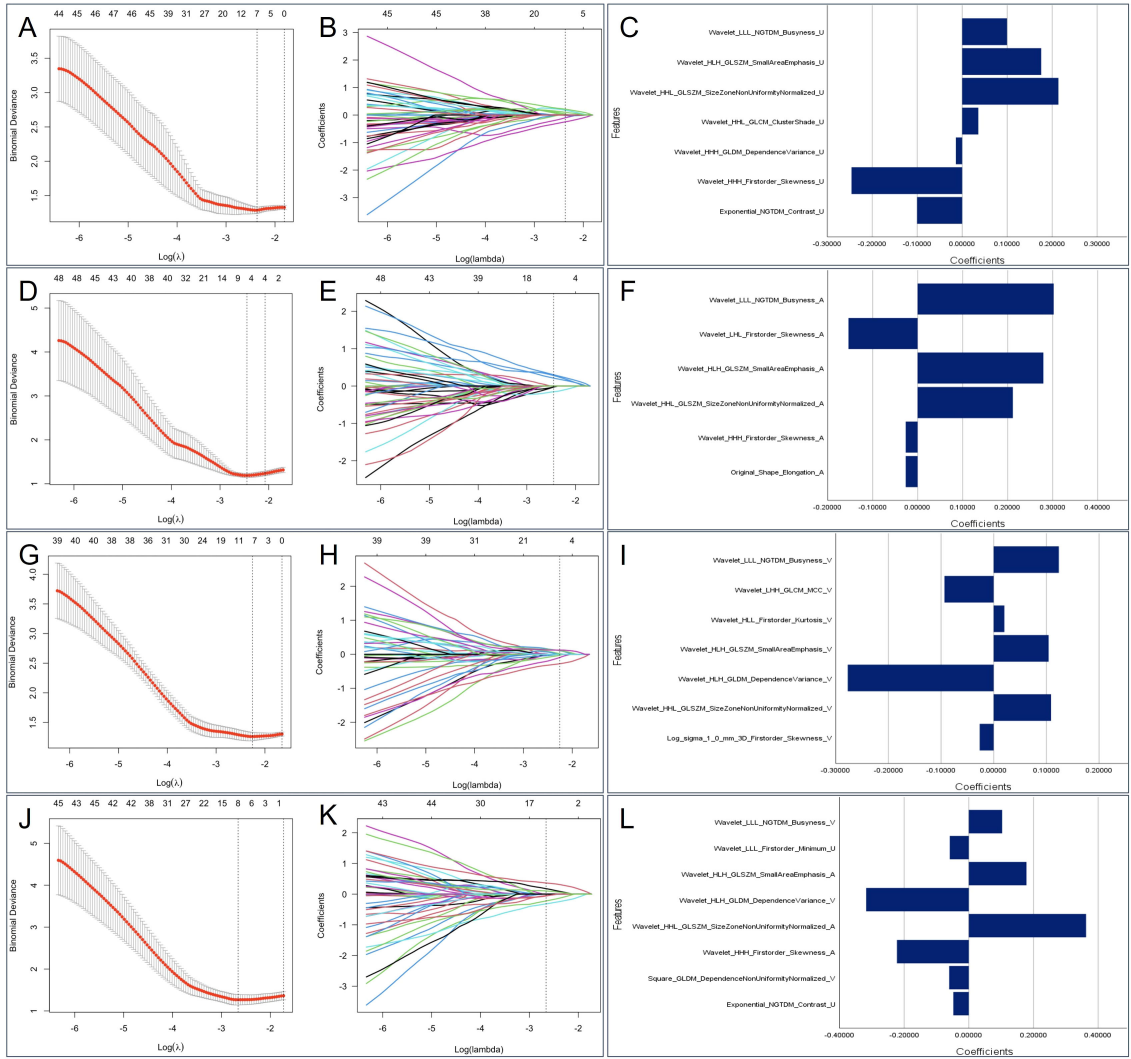
The selected radiomics features from LASSO regression. Based on minimum criteria, LASSO regression selected 7, 6, 7 and 8 radiomics features from the **(A)** unenhanced phase, **(D)** arterial phase, **(G)** venous phase, and **(J)** Triphasic (unenhanced + arterial + venous phase) CT images. The coefficient profile plots of the identified non-zero coefficients for **(B)** unenhanced phase, **(E)** arterial phase, **(H)** venous phase, and **(K)** Triphasic radiomics features were generated against the selected log λ values. The names and corresponding weighting coefficients of the selected **(C)** unenhanced phase, **(F)** arterial phase, **(I)** venous phase, and **(L)** Triphasic radiomics features. LASSO, least absolute shrinkage and selection operator.

In total, 1374 feature parameters were extracted from each ROI. The feature parameters extracted from the unenhanced phase, arterial phase, venous phase, and unenhanced + arterial + venous phase in the training group were processed by LASSO to screen out features with high generalizability. Finally, 7, 6, 7, and 8 optimal radiomics features were retained in Model U, Model A, Model V, and Model U+A+V, respectively (**Figure 3**). After excluding the repeated features, 14 radiomics features were extracted from the four radiomics models, which included one shape feature, one first-order statistical features, one GLDM feature, one NGTDM feature, and 10 high-order statistical features obtained via wavelet transform, as detailed in **Supplementary Figure S2**.

The Rad-score of each radiomics model was significantly different between the invasive and noninvasive PSPN groups, as displayed in **Table 3**. Among the three single-phase models (Model U, Model A, and Model V), Model A exhibited the best diagnostic performance, with AUCs of 0.777 and 0.769 in the training and validation sets, respectively. In addition, the radiomics combined model (model U+A+V) had AUCs of 0.857 and 0.839 in the training and validation sets, respectively. The detailed AUC results are shown in **Table 5**.

### 3.4 Performance analysis of the radiomics nomogram

**Figure 4:**
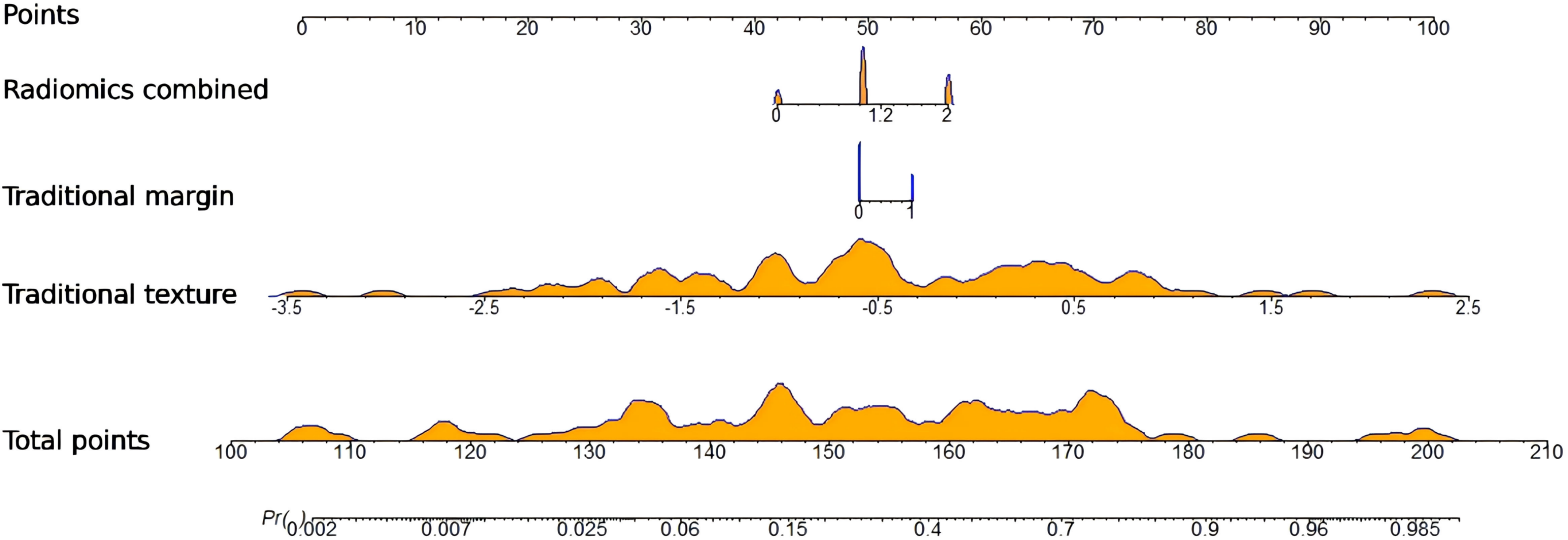
Developed radiomics nomogram for predicting invasive pancreatic solid pseudopapillary neoplasm. Dynamic nomogram for radiomics and traditional CT features: https://weiyuguo.shinyapps.io/nomogram_shinyapp/.

**Figure 5:**
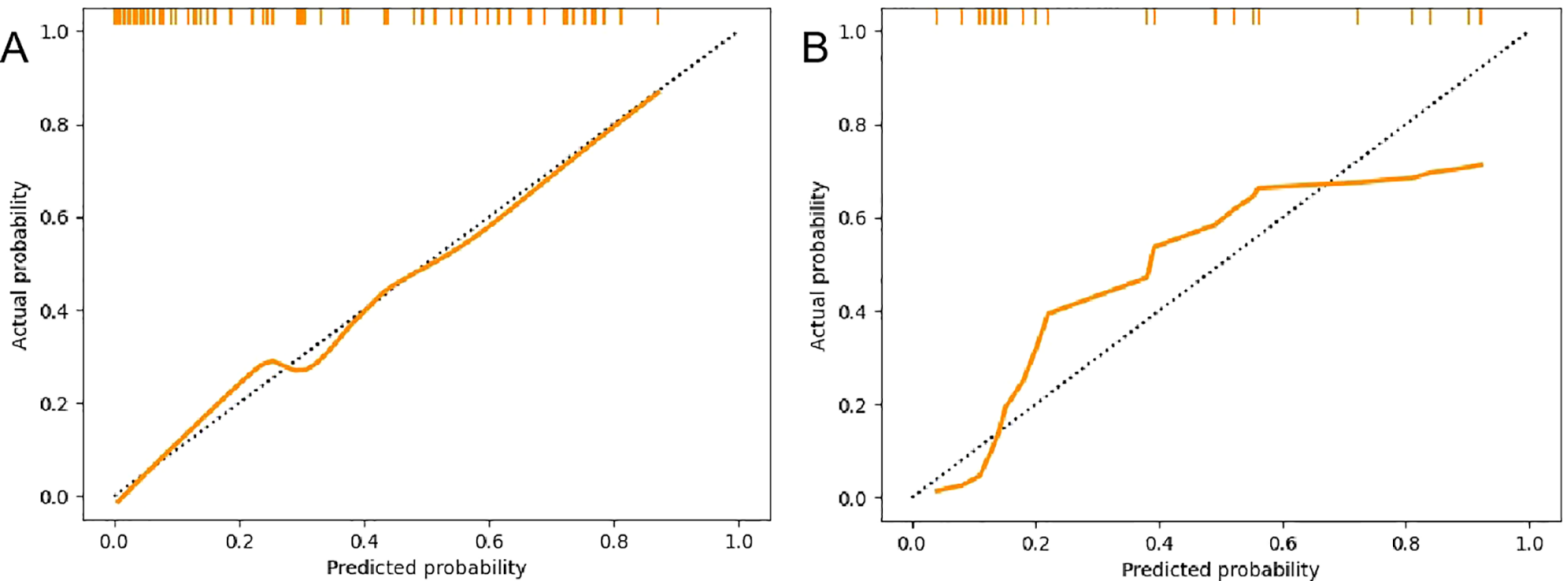
Calibration curves of the radiomics nomogram. **(A)** Calibration curves of the training set. **(B)** Calibration curves of the validation set.

**Figure 6:**
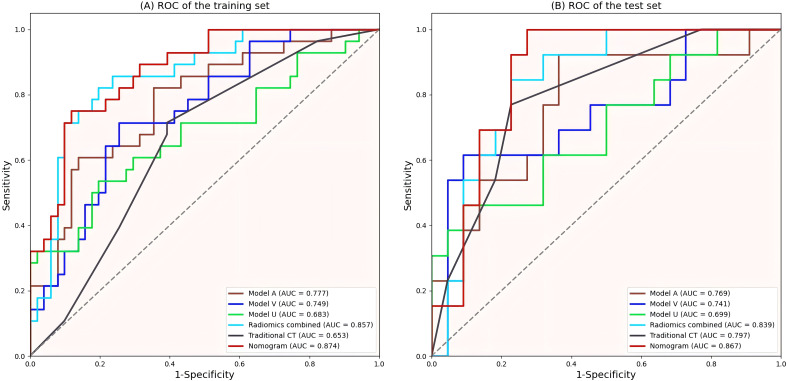
ROC curves of all models. **(A)** ROC curves of the training set. **(B)** ROC curves of the validation set. ROC, Receiver operating characteristic; AUC, area under curve.

**Figure 7:**
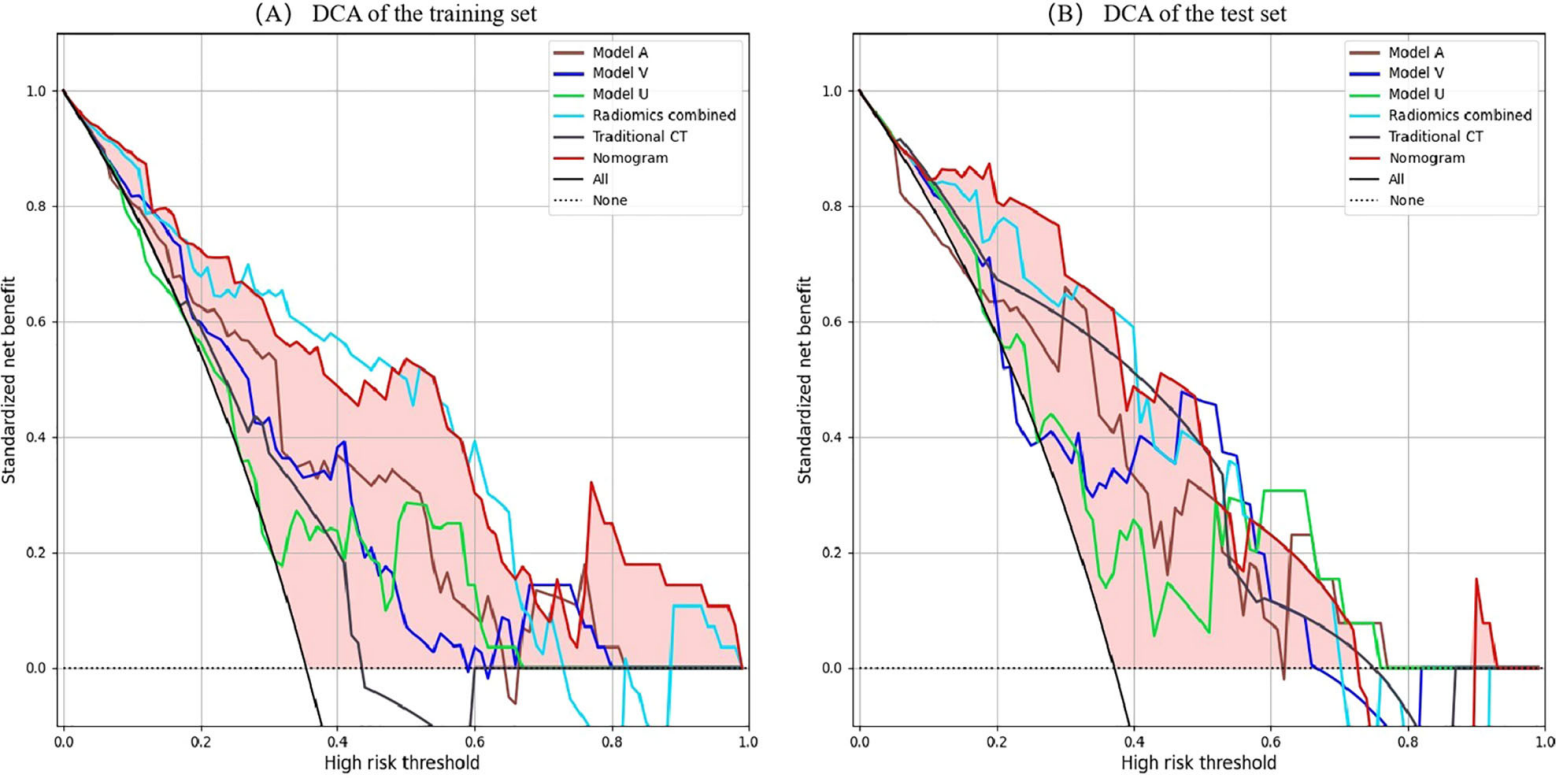
DCA of the all models. **(A)** DCA curves of the training set. **(B)** DCA curves of the validation set. X-axis represents threshold probability; Y-axis represents net benefit. All line indicates that all patients are invasive PSPN; None line indicates that all patients were non-invasive PSPN. The closer the decision curves to the black and gray curves, the lower the clinical decision net benefit of the model. DCA, decision curve analyses; PSPN, pancreatic solid pseudopapillary neoplasm.

A radiomics nomogram was constructed and calibrated on the basis of the tumor texture, margin, and Rad-score (**Figure 4**). The calibration curve and Hosmer-Lemeshow test indicated that the training (P = 0.281) and validation sets (P = 0.057) were well calibrated (**Figure 5**). The AUCs of the nomogram in the training and validation sets were 0.874 and 0.867, respectively (**Table 5**), which were better than those of the radiomics model and the traditional CT model (**Figure 6**). According to Delong’s tests, in the training set, the AUC of the radiomics nomogram was significantly different from those of Model U (P = 0.003), Model V (P = 0.018), and the traditional CT model (P < 0.001), but not significantly different from those of Model A (P = 0.103) or Model U+A+V (P = 0.399). As shown in the validation set, there was no statistically significant difference between the radiomics nomogram and Model U (P = 0.069), Model A (P = 0.318), Model V (P = 0.273), Model U+A+V (P = 0.234), or the traditional CT model (P = 0.322). According to the DCA results, the radiomics nomogram provided increased net benefit for clinical decision-making when the threshold probability was within the relevant range (**Figure 7**). We built a dynamic nomogram using the R Shiny framework that allows physicians to input radiomic features and traditional CT parameters in real time and dynamically display personalized predictions. The website is: https://weiyuguo.shinyapps.io/nomogram_shinyapp/.

The nomo-score was calculated via the following formula: Nomo−score=1.9647×Rad−score+0.5262×Marign+0.8523×Texture−1.2467 .

## 4 Discussion

In this study, predictive models incorporating radiomics and traditional CT indicators were developed to differentiate between invasive and non-invasive PSPN. Among all the models, the radiomics nomogram integrating traditional CT signs with radiomics signature demonstrated the highest diagnostic performance, with AUCs of 0.874 and 0.867 in the training and validation sets, respectively. This performance surpassed that of both the traditional CT model and the radiomics models. These findings suggest that the combined use of radiomics and traditional CT signs enhances classification accuracy, potentially aiding in clinical treatment decision-making.

Previous studies (22, 23) have shown that sex, age, tumor shape calcification, pancreatic atrophy, and pancreatic duct dilation are not significantly different between invasive and noninvasive PSPN patients, and similar results were obtained in our study. Liang et al. (24) indicated that the location of the tumor varied significantly between invasive and noninvasive PSPN, with invasive PSPN more frequently found in the pancreatic tail. However, the present study showed no statistically significant difference in tumor location. The discrepancy may be attributed to the different grouping methods used; in this study, the pancreas was divided into two groups, with the head and neck classified together and the body and tail grouped separately, which differs from the classification used in previous research. Wang et al. (8) reported that tumor size is an independent predictor of invasiveness in PSPN patients. Kim et al. (25) reported that when the tumor diameter was greater than 5 cm, the probability of presenting with invasive PSPN significantly increased (*P* = 0.022). However, to date, there is no consensus on the ability of tumor size to predict invasive PSPN. In our study, tumor size was statistically significant only in the validation set but not in the total sample, and there may be bias due to the small sample size in the validation set. Further large sample studies are needed for verification in the future. Wang et al. (8) and Rastogi et al. (26) reported no significant differences in the solid component between invasive and noninvasive PSPN, whereas in our study, invasive PSPN predominantly presented as solid or mixed solid-cystic tumors, and the solid component was significantly different (P<0.05). This finding suggests that tumor invasive behavior may be related to the extent of solid components, possibly because increased vascularity within the tumor leads to invasive growth. Moreover, the difference in tumor margins between invasive PSPN and noninvasive PSPN was statistically significant, possibly because PSPN has a richer blood supply and is more likely to have external growth, resulting in focal discontinuity of the capsule and invasion of the pancreas and surrounding adipose tissue, which results in unclear tumor boundaries.

Radiomics is an emerging discipline of high-throughput extraction of quantitative features from medical images and the use of these features to build models for clinical decision-making with the goal of improving diagnostic accuracy (27–29). In recent years, radiomics has shown good advantages in the field of medicine (30–32). However, there has been limited research utilizing multiphase CT radiomics to predict the invasiveness of PSPN; only Huang et al. (33) used dual-phase contrast-enhanced imaging to predict the invasiveness of PSPN. However, fewer cases were included in Huang’s study than in our study, and the study lacked further verification and did not explore the significance of unenhanced phases and traditional CT signs. In this study, the combination of radiomics and traditional CT signs to construct a radiomics nomogram may fill this gap.

In our study, 14 valuable radiomics features were extracted from the four radiomics models, most of which were first-order and texture features from wavelet-transformed images. The optimal features of the four models all included Wavelet_LLL_NGTDM_Busyness features. The high Busyness values in NGTDM features reflects the heterogeneity between the lesion pixel local grayscale and adjacent pixel heterogeneity. Invasive PSPN has a higher Busyness value than non-invasive PSPN. The invasive PSPN may have a more complex texture due to necrosis, neovascularization or uneven cell density, which is manifested as a higher Busyness value. The Dependence Variance of GLDM is an important feature for quantifying the heterogeneity of local gray-scale dependency in images. It is jointly influenced by cell density, interstitial proportion and vascular distribution. The variation of Dependence Variance value may be used to quantify the complex microenvironment within invasive PSPN. The size zone non-uniformity values in GLSZM features is positively correlated with the invasiveness of PSPN. High size zone non-uniformity value indicates represent a non-uniform texture, that is, high heterogeneity, and can be used as a powerful indicator to predict the invasiveness of PSPN. In addition, the skewness and kurtosis values extracted from wavelet transform images have significant weights and higher values in invasive PSPN, reflecting the complexity and heterogeneity of the cell density distribution in invasive PSPN, possibly due to rich abnormal vascular formation, changes in cell permeability, and necrosis, resulting in mixed internal tumor components and complex grayscale distributions. These findings suggest that the internal structure and heterogeneity of tumors are closely related to invasiveness. The above results indicate that radiomics features are potential noninvasive biomarkers for predicting the aggressiveness of PSPN.

Wang et al. (8) reported that CT imaging findings may help distinguish invasive PSPN from noninvasive PSPN, and the AUC was 0.77. In the current work, the diagnostic efficacy of the radiomics model was better than that of Wang’s study, indicating that radiomics contributes greatly to diagnosis. In a study by Rastogi et al. (26) in which CECT was used to predict PSPN invasiveness, invasive PSPN showed a greater degree of enhancement than noninvasive PSPN did in the delayed phase, whereas the arterial and venous phases were not significantly different. In this study, all three single-phase models were capable of predicting PSPN invasiveness, with the arterial-phase model exhibiting the highest performance. This may be attributed to the fact that the increased blood supply in invasive PSPNs is more effectively captured in arterial-phase images. Furthermore, invasive PSPNs are more prone to vascular invasion, resulting in compensatory increases in peripheral blood supply, which enhances the visibility in arterial-phase imaging. Additionally, due to the presence of mixed substances such as hyaluronic acid and collagen in the intercellular stroma, enhancement of the interstitial component may be observed during the venous phase due to contrast agent inflow, while vascular enhancement may be less pronounced. In contrast, arterial-phase images primarily show enhancement of tumor blood vessels, without significant enhancement of the interstitial component (33). These findings may indicate the predictive ability of radiomics features on the basis of the arterial phase in predicting tumor invasiveness. We subsequently combined the three phases to obtain a combined model, which significantly improved the predictive ability for PSPN invasiveness compared with single-phase models.

Radiomics is not the only determinant of diagnosis, and when radiomics is combined with other relevant data, more reliable and accurate results can be produced (34). Song et al. (35) constructed a nomogram combining age and MRI radiomics features, which was used to differentiate hypovascular nonfunctional pancreatic neuroendocrine tumors from PSPN. The AUC of the nomogram in the validation set was 0.920, which was greater than that of the radiomics model (AUC = 0.907). Gu et al. (36) demonstrated that combining clinical information with radiomics improved the differentiation of PSPN from three other conditions, namely adenocarcinoma, neuroendocrine tumors, and pancreatic cystadenomas (AUC = 0.962). Liang et al. (24) utilized radiomics features derived from T1-weighted imaging, T2-weighted imaging, diffusion-weighted imaging, and contrast-enhanced T1WI sequences, integrating them with clinical data to develop a radiomics nomogram for distinguishing between invasive and noninvasive PSPN, and the radiomics nomogram showed the best diagnostic performance (AUC = 0.808). In the present study, traditional CT indicators were combined with radiomics features to establish a radiomics nomogram, achieving an AUC of 0.867 in the validation set, surpassing both the traditional CT and radiomics models. When compared with Liang et al. (24), the proposed model showed enhanced predictive performance, suggesting that CECT may be more effective than MRI in assessing PSPN invasiveness. To validate the clinical applicability of the nomogram model, DCA was performed, indicating that within the specified threshold probability range, this model provided greater net benefit for clinical decision-making.

The primary objective of radiomics is to facilitate classification and prediction. Diagnostic and classification models in radiomics research predominantly rely on machine learning (ML) techniques, such as logistic regression analysis, random forests, support vector machines, LASSO, and linear discriminant analysis, with logistic regression being the most widely used. In this study, only the logistic regression model was employed to assess PSPN invasiveness, and the efficacy of other algorithms in similar ML tasks remains uncertain and warrants further exploration.

This study has several limitations. First, the retrospective nature of the analysis, conducted across multiple centers, may introduce selection bias in patient cohorts. Second, manual delineation of ROIs by different readers could lead to bias, potentially impacting the reliability of radiomics features. Although features with ICC > 0.75 were selected to mitigate this issue, there is a critical need for automated and accurate tumor segmentation techniques. Thirdly, This study only used 2D ROI analysis, which may ignore the spatial heterogeneity of lesions, while 3D ROI retains the three-dimensional spatial information of lesions and can extract more abundant radiomics features. In the future, we will further expand the data and discuss the advantages and disadvantages of 2D ROI and 3D ROI. Finally, due to the lack of external validation, we cannot ensure that our model has the same diagnostic performance when dealing with external datasets.

## 5 Conclusion

In conclusion, Our study shows that combining traditional CT signs with radiomics and constructing a radiomics nomogram based on multi-phase contrast-enhanced CT can effectively predict the invasiveness of PSPN, which may aid in clinical decision-making for treatment strategies.

## Data Availability

The raw data supporting the conclusions of this article will be made available by the authors, without undue reservation.
