# Supplementary material for: Prediction of solid pseudopapillary tumor invasiveness of the pancreas based on multiphase contrast-enhanced CT radiomics nomogram
Source: Front Oncol. 2025 Apr 7;15:1513193. doi: 10.3389/fonc.2025.1513193 (PMC12010104; doi:10.3389/fonc.2025.1513193)
Supplement: Supplementary file 1 [file DataSheet1.docx]

**Supplementary documents**


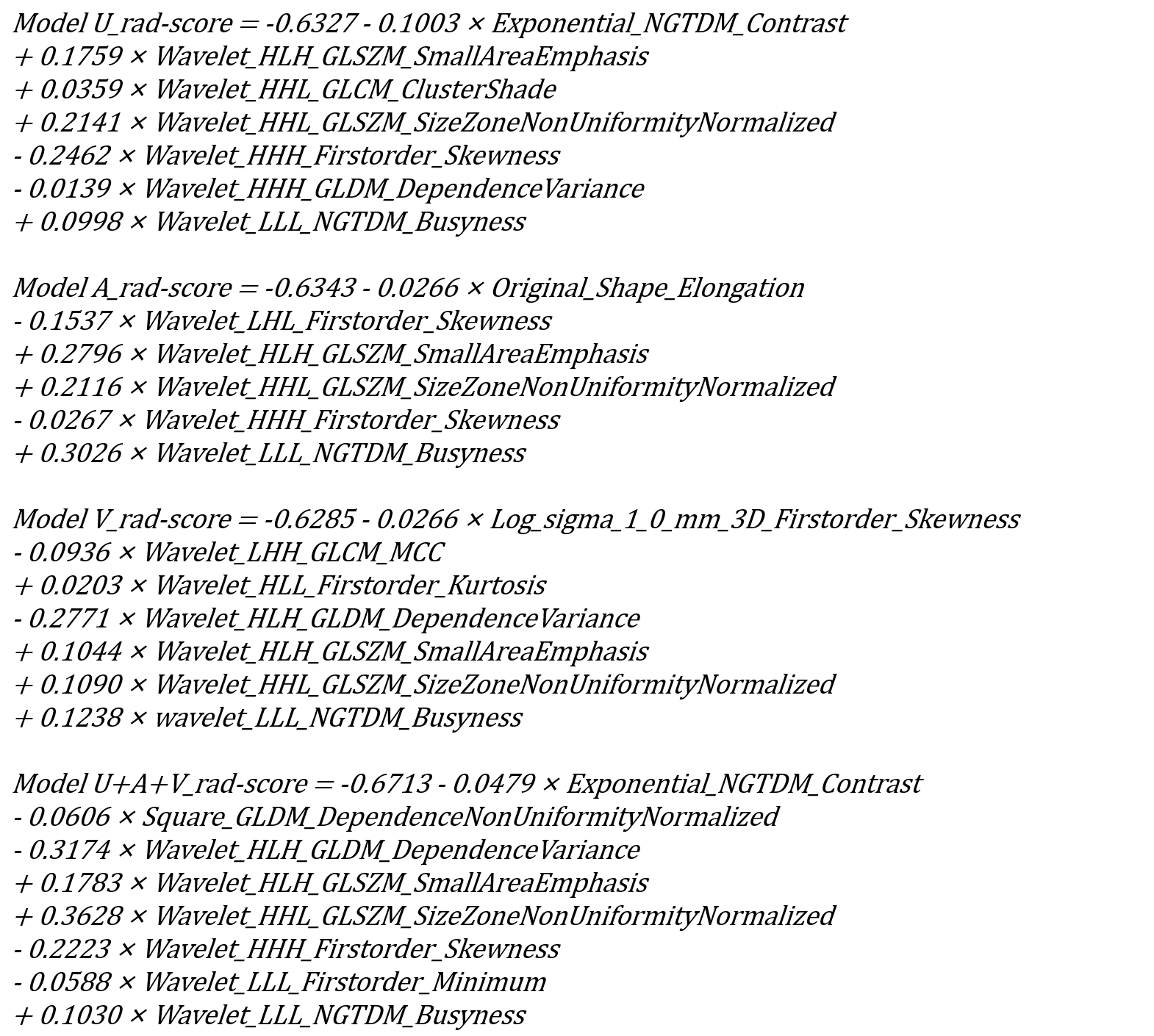


**Figure S1** Rad-score formulas for models U, A, V and U+A+V.


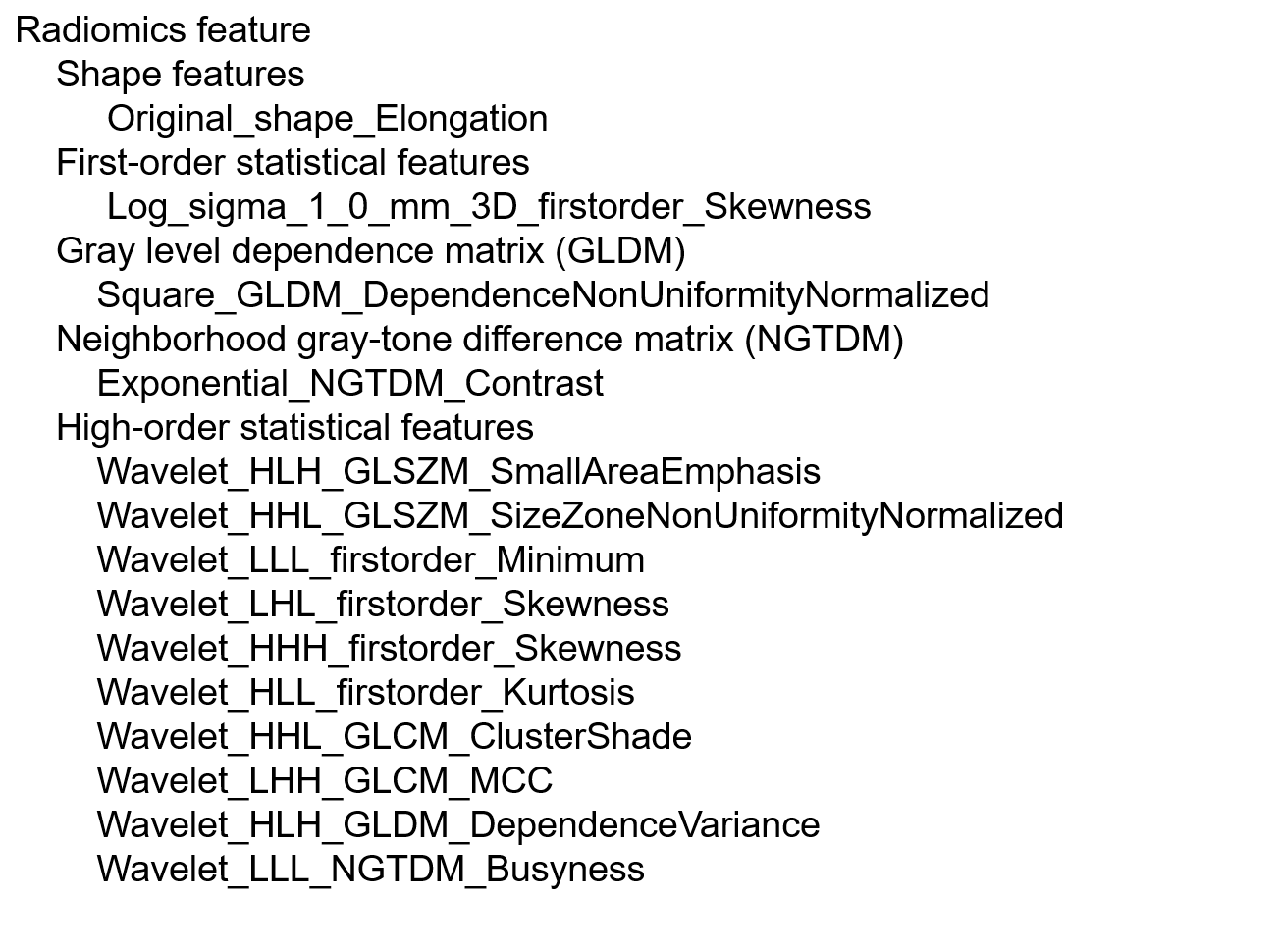


**Figure S2** Radiomics features were extracted from the four radiomics models.


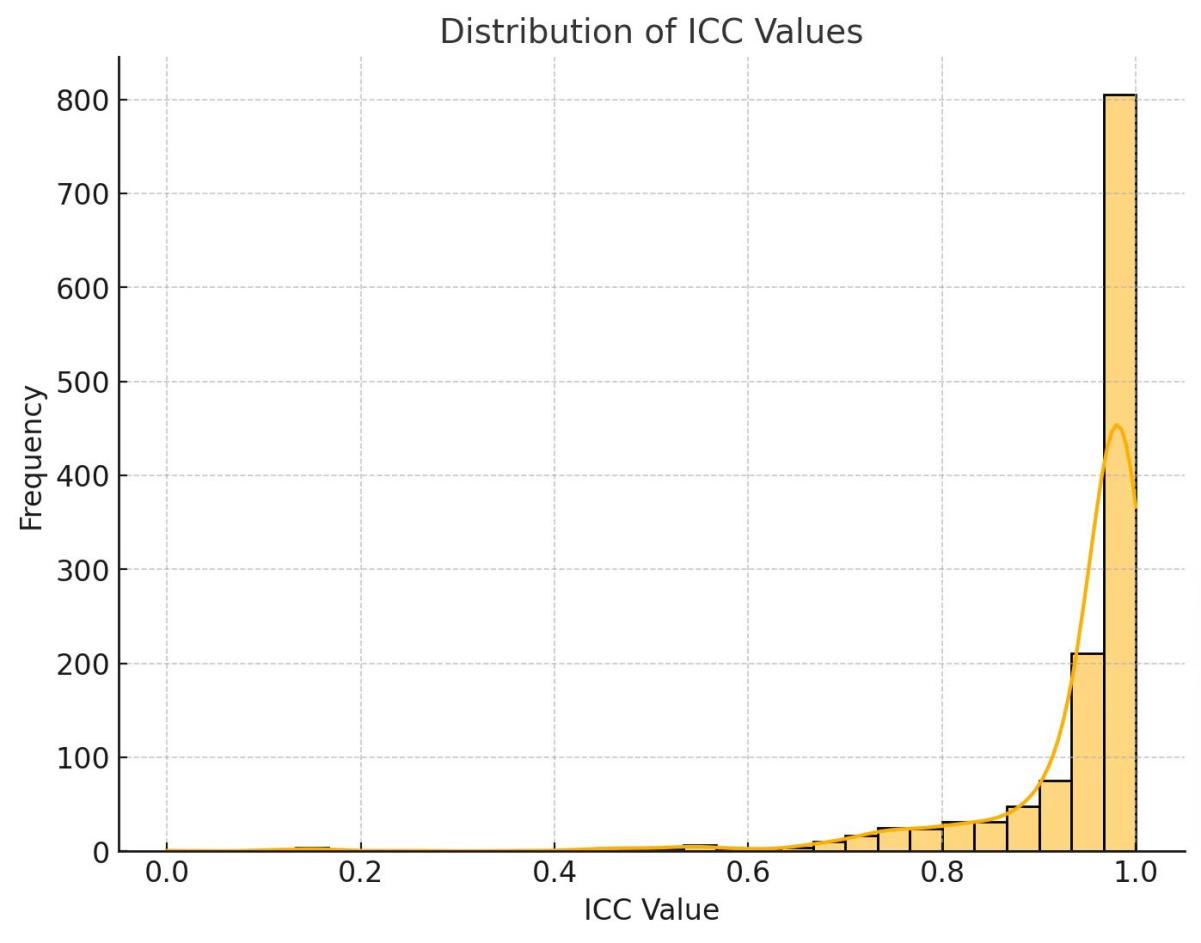


**Figure S3** Interclass correlation coefficient results of radiomic feature extraction.

Table 1 The folding performance metrics results of 5-fold cross-verification

| Fold 1 | AUC | Accuracy | Sensitivity | Specificity | PPV | NPV |
| --- | --- | --- | --- | --- | --- | --- |
| Model U | 0.8254 | 0.5626 | 0.0000 | 1.0000 | 0.0000 | 0.5625 |
| Model A | 0.7937 | 0.6250 | 0.1429 | 1.0000 | 1.0000 | 0.6000 |
| Model V | 0.7143 | 0.6250 | 0.2857 | 0.8889 | 0.6667 | 0.6154 |
| Model U+A+V | 0.9365 | 0.8750 | 0.7143 | 1.0000 | 1.0000 | 0.8182 |
| Fold 2 |  |  |  |  |  |  |
| Model U | 0.5000 | 0.6250 | 0.5000 | 0.6667 | 0.3333 | 0.8000 |
| Model A | 0.8958 | 0.8125 | 1.0000 | 0.7500 | 0.5714 | 1.0000 |
| Model V | 0.7917 | 0.8125 | 0.2500 | 1.0000 | 1.0000 | 0.8000 |
| Model U+A+V | 0.6875 | 0.7500 | 0.5000 | 0.8333 | 0.5000 | 0.8333 |
| Fold 3 |  |  |  |  |  |  |
| Model U | 0.6833 | 0.6875 | 0.1667 | 1.0000 | 1.0000 | 0.6667 |
| Model A | 0.7500 | 0.6875 | 0.3333 | 0.9000 | 0.6667 | 0.6923 |
| Model V | 0.8000 | 0.6250 | 0.3333 | 0.8000 | 0.5000 | 0.6667 |
| Model U+A+V | 0.9333 | 0.8750 | 0.6667 | 1.0000 | 1.0000 | 0.8333 |
| Fold 4 |  |  |  |  |  |  |
| Model U | 0.5167 | 0.6250 | 0.1667 | 0.9000 | 0.5000 | 0.6429 |
| Model A | 0.9167 | 0.8125 | 0.6667 | 0.9000 | 0.8000 | 0.8182 |
| Model V | 0.9500 | 0.8125 | 0.5000 | 1.0000 | 1.0000 | 0.7692 |
| Model U+A+V | 0.9167 | 0.8750 | 0.8333 | 0.9000 | 0.8333 | 0.9000 |
| Fold 5 |  |  |  |  |  |  |
| Model U | 0.9600 | 0.7333 | 0.2000 | 1.0000 | 1.0000 | 0.7143 |
| Model A | 0.5400 | 0.6667 | 0.4000 | 0.8000 | 0.5000 | 0.7273 |
| Model V | 0.4600 | 0.5333 | 0.2000 | 0.7000 | 0.2500 | 0.6364 |
| Model U+A+V | 0.8600 | 0.7333 | 0.6000 | 0.8000 | 0.6000 | 0.8000 |
| Average Results |  |  |  |  |  |  |
| Model U | 0.6971 (±0.1773) | 0.6467 (±0.0587) | 0.2067 (±0.1625) | 0.9133 (±0.1293) | 0.5667 (±0.3887) | 0.6773 (±0.0786) |
| Model A | 0.7792 (±0.1347) | 0.7208 (±0.0775) | 0.5086 (±0.2976) | 0.8700 (±0.0872) | 0.7076 (±0.1774) | 0.7676 (±0.1356) |
| Model V | 0.7432 (±0.1609) | 0.6817 (±0.1119) | 0.3138 (±0.1028) | 0.8778 (±0.1163) | 0.6833 (±0.2906) | 0.6975 (±0.0736) |
| Model U+A+V | 0.8668 (±0.0938) | 0.8217 (±0.0655) | 0.6629 (±0.1115) | 0.9067 (±0.0827) | 0.7867 (±0.2050) | 0.8370 (±0.0338) |

U, unenhanced CT; A, arterial phase CT; V, venous phase CT.
